# Comparison of Genome-Wide Association Methods in Analyses of Admixed Populations with Complex Familial Relationships

**DOI:** 10.1371/journal.pone.0088926

**Published:** 2014-03-24

**Authors:** Naveen K. Kadri, Bernt Guldbrandtsen, Peter Sørensen, Goutam Sahana

**Affiliations:** Centre for Quantitative genetics and Genomics, Department of Molecular Biology and Genetics, Aarhus University, Aarhus, Denmark; University of North Carolina, United States of America

## Abstract

Population structure is known to cause false-positive detection in association studies. We compared the power, precision, and type-I error rates of various association models in analyses of a simulated dataset with structure at the population (admixture from two populations; *P*) and family (*K*) levels. We also compared type-I error rates among models in analyses of publicly available human and dog datasets. The models corrected for none, one, or both structure levels. Correction for *K* was performed with linear mixed models incorporating familial relationships estimated from pedigrees or genetic markers. Linear models that ignored *K* were also tested. Correction for *P* was performed using principal component or structured association analysis. In analyses of simulated and real data, linear mixed models that corrected for *K* were able to control for type-I error, regardless of whether they also corrected for *P*. In contrast, correction for *P* alone in linear models was insufficient. The power and precision of linear mixed models with and without correction for *P* were similar. Furthermore, power, precision, and type-I error rate were comparable in linear mixed models incorporating pedigree and genomic relationships. In summary, in association studies using samples with both *P* and *K*, ancestries estimated using principal components or structured assignment were not sufficient to correct type-I errors. In such cases type-I errors may be controlled by use of linear mixed models with relationships derived from either pedigree or from genetic markers.

## Introduction

The power of an association study depends on the phenotypic variance explained by the causal variant, the extent of linkage disequilibrium (LD) between the causal variant and the markers, and, not least, the size of the study sample. Several recent association studies have thus focused on collecting large samples to obtain higher powers of detection [1], [2] but this practice is often associated with population stratification problems. Among the factors thought to cause lack of reproducibility in association studies reviewed [3], population stratification has probably been the most cited reason [4].

Population stratification refers to the inclusion of individuals from isolated subpopulations in the population of interest. In such a population, individuals from a subpopulation are, on average, more closely related to each other than to other individuals in the population as a whole. Population structure is common in nature. It manifests in the form of herds, colonies, and ethnic groups, and as a consequence of geographic isolation and natural or artificial selection [5]. A subtle form of stratification can also occur at the family level, especially in livestock when animals are bred in full-sib or half-sib families [6].

Genetic association studies aim to correlate differences in trait level with differences in genotypes at a tested marker. This correlation is assumed to arise from the co-occurrence of the marker with the quantitative trait locus (QTL), i.e., LD. The presence of LD is usually equated with physical linkage. However, in a stratified population with differences at the trait level, markers with different frequencies across populations will also correlate with trait levels. Thus the use of a structured population in an association study may yield false associations due to differences in allele frequencies among subpopulations [7].

Examination of an unstratified sample is thus optimal in an association study [5]. However, combining samples from multiple populations is often necessary to increase the power of detection. In some cases, study of a structured population may be advantageous. Markers tightly linked to causal variants achieve similar significance in tests of association, making it difficult to distinguish them from the causal variant. In such cases, the use of a structured sample may enable precise localization of the causal variant, as a marker in strong LD with the causal variant in one population may not be in strong LD in the other [8]. Thus, the use of statistical methods that correct for stratification at the population and family level, especially in livestock are important for the present and future association studies that aim to genotype large samples.

Several methods, such as structured analysis (SA) [9], principal component analysis (PCA) [10] and the use of linear mixed models (LMM) [5], have been reported to be useful in correcting for stratification in genome-wide association (GWA) samples. It has been noted that correcting for stratification is more challenging when family structure is present along with population stratification [11]. In this study, we simulated an admixed population (variable ancestry from two populations) with familial relatedness as generally observed in commercial cattle populations and compared the power, precision and false-positive detection rates of analyses using the SA, PCA, and LMM approaches. We also applied these methods to two publicly available real datasets and compared the incidence of false-positive associations.

## Materials and Methods

The study uses a simulated dataset and 2 publicly available datasets, so no ethical approval was required.

### Simulation Study

**Figure 1:**
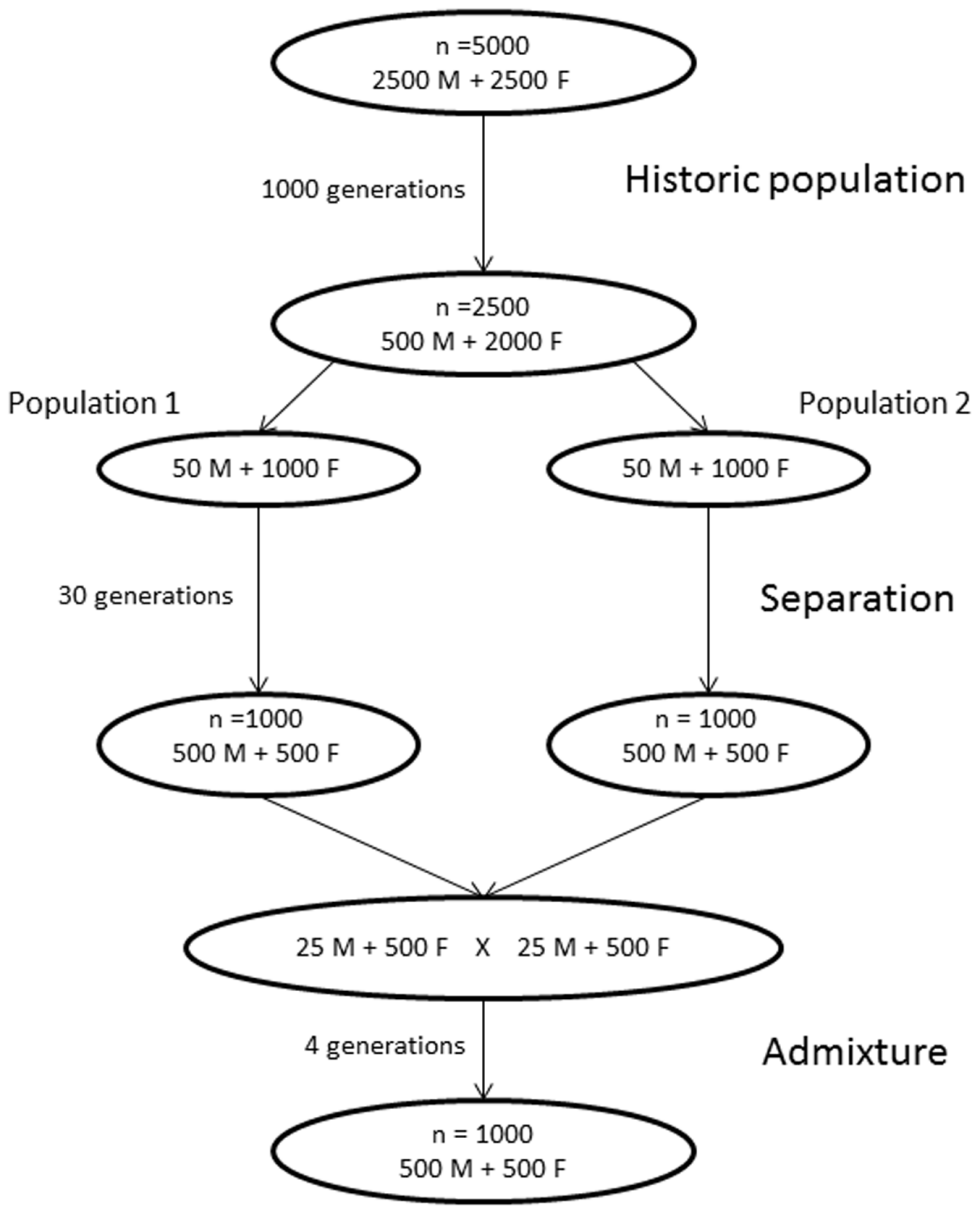
Schematic representation of the simulation.

An admixed population (Fig. 1) was simulated using the QMSim software package [12]. First, an historic population (HP) was simulated to generate the initial linkage disequilibrium (LD) and to allow for mutation (2.5×10^−3^/locus/generation) and drift. The HP consisted of 5,000 individuals that were randomly mated for 1,000 generations. The genome was assumed to have 20 chromosomes of 100 cM each with 40,000 evenly distributed bi-allelic SNP markers with equal frequencies (0.5) for the two alleles in the base population. Only two QTLs with two equally frequent alleles were simulated, both located on chromosome 1 at 20 cM and 80 cM. The QTL mutation rate was 2.5×10^−5^/generation. The number of recombination per Morgan (per chromosome) was sampled from a Poisson distribution of mean = 1 and the cross-overs were randomly placed on the chromosomes. QTL effects were introduced in the last generation of the HP for a trait (mean = 0, variance = 1) with a heritability of 0.3. The allelic effects were sampled from a gamma distribution with a shape parameter of 0.4 as implemented in QMSim [12], so that the two QTLs together explained 10% of phenotypic variance. The remaining genetic variance was simulated as a polygenic effect. After 1,000 generations, the population size was reduced to 2,500 individuals (2,000 females, 500 males).

Next, two populations were generated from the HP by mating two separate parent groups in isolation. The parent groups (1,000 females, 50 males each) were randomly selected from the last generation of the HP. Each sire was mated to 20 dams, with each mating producing one offspring. This procedure yielded a total of 1,000 offspring with an equal sex ratio (500 males, 500 females) from each parent group. Fifty randomly selected male offspring from each group were used as sires for the next generation, whereas all female offspring along with 500 randomly selected dams from the previous generation were used as dams for the next generation. This mating scheme was continued for 30 generations.

**Figure 2:**
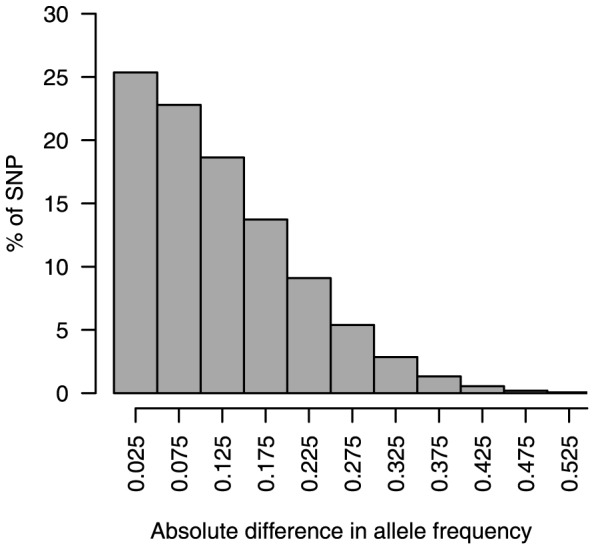
Absolute differences in allele frequencies of single-nucleotide polymorphism (SNP) markers between populations (100 replicates).

**Figure 3:**
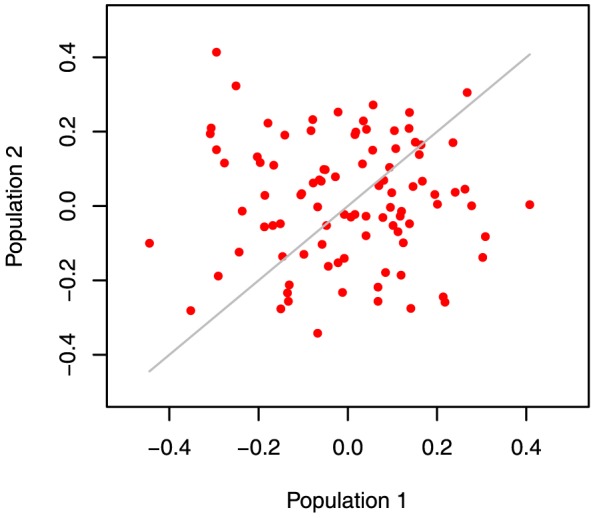
Differences in mean phenotypes of two populations after separation for 30 generations (100 replicates).

After 30 generations, the average genetic distance between populations in 100 replicates, estimated as mean *F_st_* from all 40,000 SNP markers, was 0.025 [standard deviation (SD) = 0.0006]. The allele frequencies of the markers (Fig. 2) and mean phenotype levels (Fig. 3) differed between populations.

**Figure 4:**
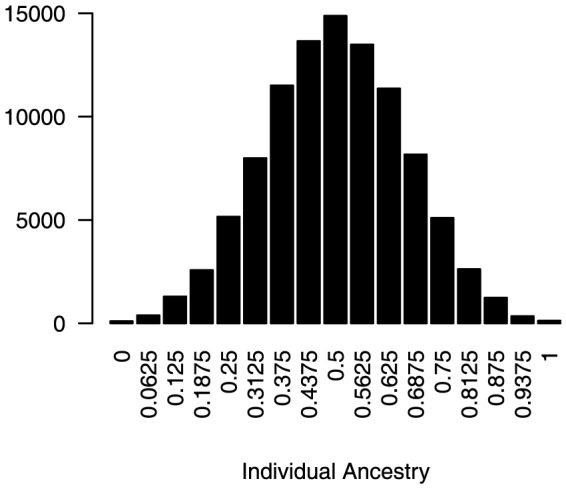
Distribution of individuals' ancestries after admixture (100 replicates).

In the next step of the simulation, individuals from two isolated populations were mated to yield an admixed population. Twenty-five males and 500 females were selected from each population to serve as parents. These individuals were randomly mated for four generations to yield an admixed population of 1,000 individuals (500 males, 500 females), following the mating scheme described above. After admixture, QTLs in the last generation explained 0–13% of the total phenotypic variance. The individuals' ancestries, calculated as average genetic contributions from one parent population to individuals in the admixed population over four generations, are presented in Figure 4. Data on phenotypes for these 1,000 samples, along with their genotypes for 40,000 SNP markers, were used in our association analyses.

Chromosome 1, which contained two simulated QTLs, was used to assess the power and precision of the models and five chromosomes (chromosomes 2–6) without simulated QTLs (null chromosomes) were used to assess the false-positive rate (type-I error). A total of 100 replications were used to compare the models.

### Publicly Available Real Data

In addition to the simulated datasets, the models for GWA studies were applied to the following two publicly available datasets to assess their ability to control type-I error.

#### Human GOLDN Dataset

This human stature dataset was collected from 1,315 European American individuals genotyped for 637 genetic markers as part of the Genetics of Lipid Lowering Drugs and Diet Network (GOLDN) study [13], [14]. Individuals' height, sex, and age were recorded. Previous studies have found no significant population structure in this sample and no significant association between height and any of these genotyped markers.

#### Dog Data on Hip Dysplasia

This dataset contains hip dysplasia data from 292 dogs of two breeds (Labrador retriever and greyhound) and their crosses (F_1_, F_2_, and backcrosses). The samples were genotyped for 23,500 SNPs and hip dysplasia was measured using the Norberg angle [15]. The relationship matrix was estimated from pedigree. The ancestors of each dog were traced back as far as possible. We used 1,000 randomly chosen markers assumed to be unlinked to the variants controlling hip dysplasia [14].

### Association Models

The allele substitution effect (*b*) of an SNP was estimated by successively fitting the marker in the following models. The simulated data contained two confounding factors: admixture (*P*) and familial relatedness (*K*). The models compared corrected for none, one, or both factors.

The significance of estimated *b* was then tested against the null hypothesis (*b* = 0) using a *t*-test with Bonferroni correction for multiple testing, at a significance level of *α* = 0.05. All analyses were carried out with the DMU software package [16].

1. **Linear Model (LM).** A linear model (LM) ignoring *K* and *P* was used to estimate the allele substitution effect:
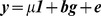
where 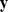 was a vector of phenotypes, 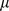 was an overall mean, 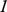 was a vector of ones, 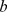 was the allele substitution effect of the SNP, 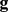 was a vector of additively coded (0, 1 and 2) SNP genotypes and 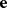 was a vector of random residuals with normal distribution 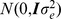, where 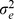 is the error variance and 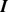 is the identity matrix.
2. **Linear Mixed Model Including Pedigree-Based Relationship (LMMped).** An LMM accounting only for *K* was used to test the associations of single SNPs with the phenotype:
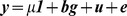
The terms in the model are similar to those in the LM, with the addition of random polygenic effects (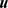). K was included in the model as the variance-covariance structure (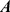) of 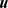, estimated from the pedigree relationships. 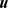 was assumed to be multivariate normally distributed 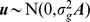, where 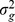 is the genetic variance and 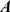 is the additive genetic relationship matrix derived from pedigree records for the last three generations.
3. **Linear Mixed Model Including Genomic Relationship (LMMgmat).** This LMM was similar to the LMMped accounting for *K*, differing only in the estimation of the variance-covariance matrix of 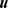. Instead of a pedigree-based relationship matrix (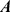), genome-wide SNP markers were used to estimate the genomic relationship matrix (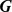) [17]. 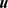 was assumed to be multivariate normally distributed 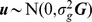.
4. **Structured Association.** A model based on the clustering method of the STRUCTURE software package [9] was used to correct for *P*. One thousand markers evenly distributed over the genome were selected and individuals were assigned to two clusters (*k* = 2) in a structure linkage model [18]. Admixture was assumed and partial membership to a cluster was allowed. The membership of an individual in a cluster (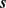, varying continuously between 0 and 1) was used as a covariate in the following models to correct for *P*.
  1. **4a) Linear Model with STRUCTURE (LMstr).** This model was similar to the LM, with additional fixed regression (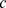) of the phenotype on 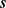 to account only for P while ignoring K:
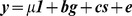

  2. **4b) Linear Mixed Model with STRUCTURE (LMMstr).** This model was similar to LMMped, with the addition of fixed regression of the phenotype on 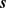 to correct for K and P:
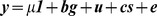

5. **Principal Component Analysis (PCA).** PCA was performed to correct for *P* in the sample. Principal components (PCs) for the covariance matrix, estimated from all 40,000 SNP markers, were calculated. SNP genotypes and phenotypes were corrected for the first two PCs, as described elsewhere [10].
  In the simulated datasets on an average the top two PCs explained 2.23% of the total variance in the range of 2.05 to 2.62. Although the fraction of the total variance explained was low, in most cases one of the first two PCs for the simulated datasets (Fig. S1) or the first PC for the dog dataset (Fig. S2) showed the strongest correlations with known ancestries and separated samples on ancestries. We also found that in most of the cases the correlation between PCs and known ancestries decayed rapidly beyond the first two PCs (Fig. S3). Thus, the first two PCs were used to correct for *P*. Although PCs did not separate the human samples, two PCs were used to correct the genotypes and phenotypes for comparison. The corrected genotypes (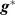) and phenotypes (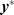) were fitted in the following models to correct for *P*.
  1. **5a) Principal Component Analysis in a Linear Model (LMpca).** Here, 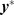 and 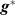 were fitted in the LM to account only for *P* while ignoring *K*:
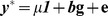

  2. **5b) Principal Component Analysis in a Linear Mixed Model (LMMpca).** Here, 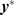 and 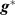 were fitted in LMMped to correct for *K* and *P*:
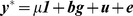

### Comparison of the Models

#### Type-I Error

*Simulated Data*. SNPs on five null chromosomes (with no simulated QTL) were used to assess the type-I error rates of all described models. A total of 1,000,000 SNPs over 100 replicates were fitted into the models to estimate *b*. The distribution of −log_10_ *p*-values of the *t*-test was plotted against the null (uniform) distribution in a quantile-quantile (QQ) plot. Deviation from the null distribution was used to assess the type-I error rate. In addition, the number of observed significant SNPs on null chromosomes was compared with the expected number at different significance levels.

#### Real Data

The human data were analyzed with LM, LMMped, LMMpca (first two PCs), and LMMstr (*k* = 2), with the inclusion of sex, age, and squared age as covariates [14]. Previous studies have reported no significant association between the SNPs and height. The deviation of −log_10_ *p*-values of the *t*-test from the expected uniform distribution in a QQ plot was used to assess the type-I error rate.

The data on hip dysplasia in dogs were fitted to the LM, LMMped, LMMpca (first two PCs), LMMstr (*k* = 2), and LMMpedB, a model similar to LMMped but with the addition of breed (as proportion of Labrador retriever) as a fixed regression. The 1,000 randomly chosen markers were assumed to be unassociated with the causal variants for hip dysplasia. The distribution of −log_10_ *p*-values of the test of association was compared with the expected distribution under the assumption of no association in a QQ plot. Deviation from the expected distribution was used to assess the type-I error rate.

#### Power and Precision

The power of the models to detect the QTL was assessed from chromosome 1, with 2 simulated QTL, i.e., for 199 QTLs over 100 replicates (one QTL fixed for an allele was left out of the analysis). A QTL was considered detected when an SNP within 1 cM of the simulated QTL showed significance after Bonferroni correction (for 12,000 SNPs tested on six chromosomes) at α = 0.05. The absolute distance between the detected and simulated QTL positions was calculated to ascertain the precision of the methods in locating the QTL. Power and precision were estimated separately for small effect (explaining <5% of 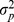) and large effect QTL (explaining >5% of 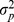).

## Results

### Results from the simulation study

#### Type-I Error

**Figure 5:**
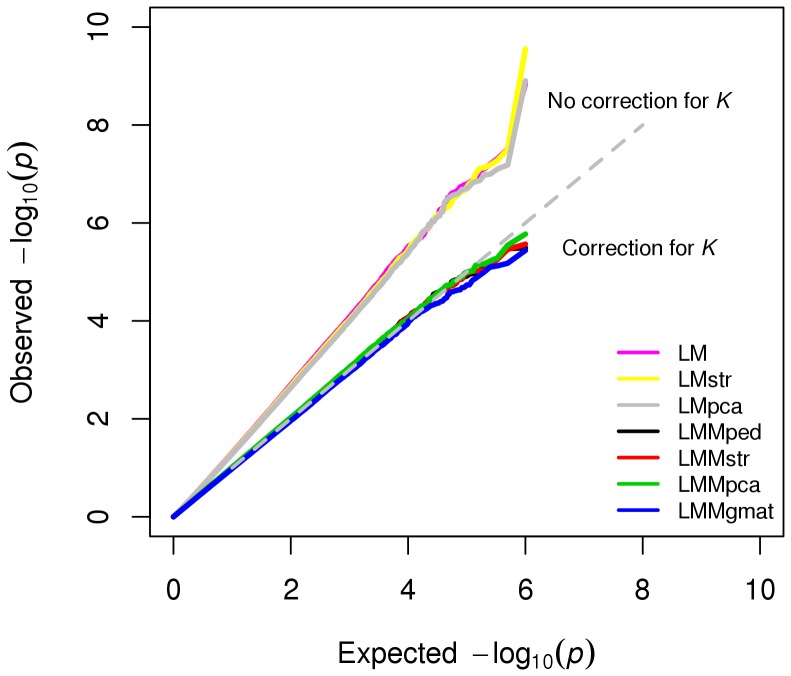
Quantile-quantile plot of −log_10_ *p*-values for association tests using different models in the simulated dataset.

The distributions of −log_10_ *p*-valuesfor the SNPs on the five null chromosomes were compared with expected uniform distributions (Fig. 5). In general, type-I error rates were lower in LMMs, where familial relationship (K) was used to model the covariance structure of the random individual effect than in LMs (which did not account for *K*). Among LMMs, LMMpca closely followed the expected type-I error rate, whereas LMMgmat was the most conservative, showing a lower than expected type-I error rate.

The average number of significant SNPs on the null chromosomes is compared with expected numbers under the null hypothesis at different significance levels in Table 1. At a Bonferroni-corrected significance level, α = 0.05 (corrected for 10,000 SNPs tested on five chromosomes) LMs that did not account for *K* (LM, LMstr, and LMpca) showed significantly higher false-positive results than expected (*p*<0.01), whereas LMMs that corrected for *K* (LMMped, LMMstr, LMMpca, and LMMgmat) showed better control of false-positive results.

**Table 1:** Average number of significant single-nucleotide polymorphisms (SNPs; 100 replicates) in five chromosomes (10,000 SNPs) without simulated quantitative trait loci.

Model		Significance level			
	Correction for	0.05	0.005	0.0005	0.000005
LMMped	*K*	514.73* (7.83)	53* (1.7)	5.5 (0.4)	0.02 (0.014)
LMMstr	*P*+*K*	511.88 (7.9)	51.97 (1.6)	5.64* (0.4)	0.02 (0.014)
LMMpca	*P*+*K*	520.89** (7.6)	53.92** (1.6)	5.88** (0.4)	0.02 (0.014)
LMMgmat	*K*	472.15 (5.7)	44.92 (1.2)	4.45 (0.3)	0.01 (0.01)
LM	-	1014.51** (18.9)	190.2** (6.8)	36.93** (2.0)	1.43** (0.17)
LMstr	*P*	998.22** (17.8)	184.33** (6.4)	34.78** (1.8)	1.34** (0.16)
LMpca	*P*	968.01** (18.7)	175.27** (6.5)	32.01** (1.7)	1.17** (0.16)
Expected false-positive associations		500	50	5	0.05

Standard errors are given in parentheses. The average number of significant SNPs (S_obs_) in 100 replicates was compared with the expected number (S_exp_) at different significance levels using *t*-tests. (H_0_: S_obs_ = S_exp_; H_1_: S_obs_>S_exp_; *p<0.05, **p<0.01). Significance level of 0.000005 corresponds to a nominal significance level of 0.05 after Bonferroni correction for 10000 tests.

*LMMped = Linear Mixed Model Including Pedigree-Based Relationship, LMMstr = Linear Mixed Model with STRUCTURE, LMMpca = Principal Component Analysis in a Linear Mixed Model, LMMgmat = Linear Mixed Model Including Genomic Relationship, LM = Linear Model, LMstr = Linear Model with STRUCTURE, LMpca = Principal Component Analysis in a Linear Model, P = admixture, K = Familial relationships.*

#### Power

**Figure 6:**
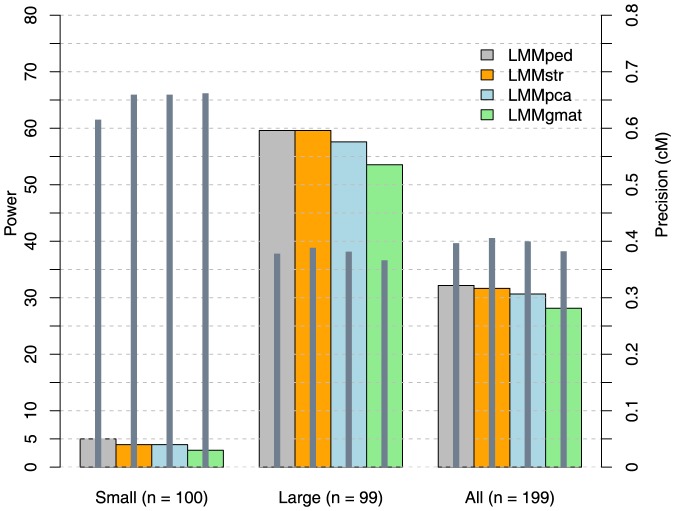
The power [% of quantitative trait loci (QTLs) detected] and precision (absolute distance between simulated and detected QTL; gray bars) of the models in QTL localization.

The power of the models to detect two simulated QTLs on chromosome 1 using 100 replicates with 199 QTLs is summarized in Figure 6. Power was compared only among LMMs because the LMs had very high false-positive detection rates (Fig. 5). No LMM had the power to detect small-effect (<5% of 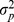) QTLs. The power was ∼5%, at the level expected with *α* = 0.05. In contrast, all models tested showed 53–60% power to detect large-effect (explaining >5% of 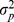) QTLs. LMMped and LMMstr had the highest powers (59.6%), followed by LMMpca (57.6%) and LMMgmat (53.5%), although this difference was not significant [χ2 (3, *n* = 99) = 0.9925; *p* = 0.8031]. Overall, LMMped had the highest power, detecting 32% of simulated QTLs, followed by LMMstr (31.7%), LMMpca (30.7%), and LMMgmat (28.1%); however, this difference was also not significant [χ^2^ (3, *n* = 199) = 0.8983; *p* = 0.8258].

Out of the total 199 simulated QTLs, 53 were detected by all the four models, the majority (50) of which being large effect QTL (mean genetic variance = 0.087, SD = 0.02). On the other hand 133 QTLs were not detected by any of the models, majority (95) of them being small effect QTL (mean genetic variance = 0.031, SD = 0.032).

#### Precision

The precision of the models, measured as the absolute distance (cM) between the positions of simulated and detected QTLs, is described in Figure 6 and Table 2. All four methods compared showed greater precision for the localization of large-effect compared with small-effect QTLs. On average, large-effect QTLs were localized within 0.36–0.39 cM of the simulated QTL positions, whereas small-effect QTLs were localized within 0.62–0.66 cM. LMMped was most precise (±0.62 cM) for small-effect QTLs, and LMMgmat was most precise (±0.36 cM) for large-effect QTLs.

**Table 2:** Absolute error (cM) in quantitative trait loci localization.

	Small effect	Large effect	All
LMMped	0.62	0.38	0.40
LMMstr	0.66	0.39	0.41
LMMpca	0.66	0.38	0.40
LMMgmat	0.66	0.37	0.38

Precision is given as the absolute genetic distance between simulated and detected quantitative trait loci (±1 cM).

*LMMped = Linear Mixed Model Including Pedigree-Based Relationship, LMMpca = Principal Component Analysis in a Linear Mixed Model, LMMstr = Linear Mixed Model with STRUCTURE, LMMgmat = Linear Mixed Model Including Genomic Relationship.*

### Results from the Published Data

#### Human GOLDN Dataset

**Figure 7:**
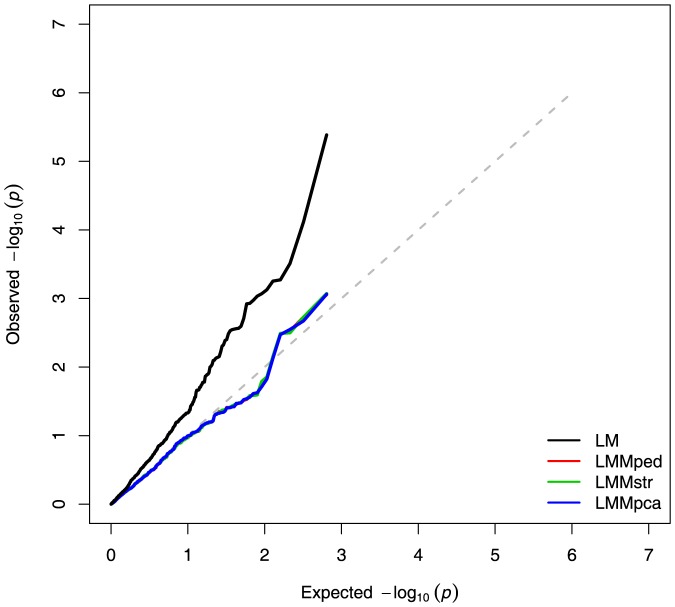
Quantile-quantile plot of −log_10_ *p*-values for association tests of the human GOLDN dataset using different models.

For the Human GOLDN dataset, type-I error was compared among the LM, LMMped, LMMstr, and LMMpca. The distribution of test statistic is given in a QQ plot (Figure 7). The LM without correction for *K* and *P* showed the highest false-positive rate. The three LMMs (LMMped, LMMstr, and LMMpca), showed similar distributions of −log10 *p*-values, which were slightly higher than expected.

#### Dog Data on Hip Dysplasia

**Figure 8:**
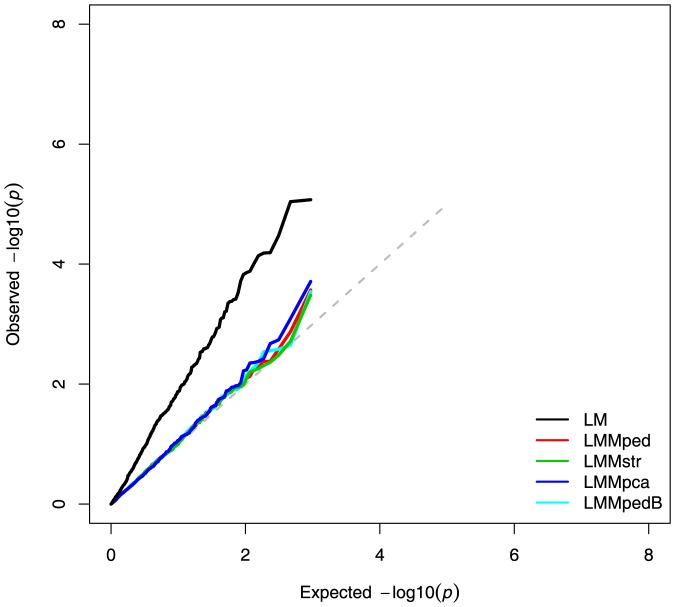
Quantile-quantile plot of −log_10_ *p*-values for association tests of the dog hip dysplasia dataset using different models.

The dog data were analyzed using the LM, LMMped, LMMpedB, LMMstr, and LMMpca. The type-I error rates for these methods are compared in a QQ plot in Figure 8. The LM showed the highest rate of type-I errors, and this rate was comparable among LMMs, with LMMpedB and LMMstr showing the lowest type-I error rates.

## Discussion

We compared the power, precision and false-positive association rates of several GWA models in analyses of simulated admixed data with familial relationships. LMs that did not model *K* (LM, LMstr, and LMpca) showed higher rates of false-positive associations than did LMMs accounting for *K* (Fig. 5, Table 1). Regardless of whether they corrected for population stratification, LMMs controlled for false-positive results at the nominal level. This is probably due to the capture of the ancestry of individuals by the variance-covariance structure modeled into the random polygenic effect, as previously shown [5]. The results of analyses of publicly available human and dog data also show that LMMped was comparable to LMMstr and LMMpca in controlling for type-I errors (Figs. 7 and 8). Thus, in a sample with structure at the family and population levels, correction for *K* alone might be sufficient to control for false-positive associations arising from population structure.

In contrast, correction for *P* alone in the LMpca and LMstr was insufficient to control for false-positive associations. This is probably because STRUCTURE [9] and PCA [10] corrected for *P*, but subtle admixture at the family level was not captured by STRUCTURE (*k* = 2) and LMpca (two PCs). Thus our findings indicate that correction for structure at the population level alone is not sufficient in samples with structure at the population and family levels. In such samples, LMMs can be effectively used to control for false-positive results.

Among LMMs, LMMgmat was the most conservative and consequently suffered a slight loss of power. In this model, the relationship matrix (G) was estimated from the whole genome, including markers linked to the simulated QTL. This probably led to correction of part of the QTL effects in the data, thereby reducing the model's power to detect QTLs.

The compared models had powers of ∼60% in detecting large-effect QTLs. In our simulation, the same set of QTLs were segregating in the two ancestral populations, which might not accurately reflect the situation in real populations, where subpopulations may have different causal variants affecting the same trait, thereby reducing the power to detect such QTLs. Thus, our power results likely reflect the upper boundary of detection power in structured populations for QTLs with similar effects using the same sample size. In our study, none of the tested methods had the power to detect small-effect QTLs in the simulated dataset. This finding might be explained by a lower average LD (*r* ^2^ = 0.07) between adjacent markers and a similar LD between markers and the QTL. Increasing the marker density and sample size might increase the power to detect small-effect QTLs in such a dataset.

It should also be noted that the LD levels in the dataset is defined by the specific parameters (mutation rate, recombination rate and type of mating) used in the simulation. The mutation rate for markers assumed in this study was higher than what is observed in real life (e.g. 10^−8^ per base per generation in human, The 1000 Genomes Project Consortium. 2010 [21]). However, we had only 2000 marker per chromosome (100 cM≈100 MB). Therefore, the mutation rate considered in the study reflects the chance of having a mutation for a genomic region the SNP represents. Altering the genetic parameters may change the LD structure; however we do not expect a change in the relative ranking of the methods in the power of QTL detection; the same is true for the type-I error rate. Moreover, we use chromosomes devoid of the simulated QTL to assess the type-I error rate; hence the false associations arise only due to stratification and not because of the LD between markers and the QTL. Our study focuses on quantitative trait, but the findings can be generalized to case-control data as reported by Kang et al., 2010 [19]; who found variance components based method to be effective in correcting for stratification.

The models showed comparable precision in QTL localization. Large-effect QTLs were localized with higher precision than were small-effect QTLs. This result contrasts with that of MacLeod et al. [20], who found that small-effect QTLs were mapped more precisely than large-effect QTLs. This difference is probably due to sampling bias, as the models in our study had no/very low power to detect small-effect QTLs, but ∼60% power for large-effect QTLs; this situation allowed comparison of only a few observations.

In our simulation, we found that in most cases (86) the first principal component captures the genetic variation due to population stratification (correlation of 0.77 with ancestry; see Fig. S1). However, in nine cases the second PC, in three cases the third PC and in two cases the fourth PC had showed the highest correlation with the ancestries (0.67, 0.58, and 0.52, respectively). These observations suggest that in a dataset with structure at both population and family level, the latter may account for more genetic variation than the former. Therefore correction for only the first two PCs might not always be sufficient to correct for population stratification. It may thus be important to find out which PCs explain the cryptic relationship better in each dataset and include those to correct population stratification.

Our findings show that LMM approaches accounting for *K* are able to correct for structure at the family and population levels. We also found that the power and type-I error rate of LMMgmat with *G* were comparable to those of LMMped with *A*. Thus, LMMs may be used to correct for structure at the family and population levels by including *K* estimated from pedigree or genetic markers.

## Supporting Information

Figure S1 **Top two axes of variation in the simulated datashowsseparation of samples along the top two axes of variation in 4 replicates of simulated data.** The samples are color coded with the individual ancestry (IA).Replicates i and iii show separation along the first axis whereas replicates ii and iv show separation along the second axis. Over 100 replicates, in 86 cases the first PC had the highest correlation (mean of 0.77) with the ancestries, in 9 cases with the second PC (0.67), in 3 cases with third PC (0.58) and in 2 cases with the fourth PC (0.52).
(EPS)

Figure S2 **Top two axes of variation in the Dog hip dysplasia datashows separation of Dog breeds, Labrador (L), Greyhound (G), and crosses along the top 2 PCs.** The first PC had a correlation of 0.91 with the breed proportions.
(EPS)

Figure S3 **Correlation between the top 20 PCs and the known ancestries in the simulated dataset (100 replicates).**
(EPS)
